# Astaxanthin suppresses hepatocellular carcinoma via targeting Wnt/Β-catenin pathway: Experimental study on chemically induced HCC in rats

**DOI:** 10.1038/s41598-026-45680-1

**Published:** 2026-04-21

**Authors:** Mona A. Kortam, Marwa Sh. Ismail, Maher A. Kamel, Noha A. El-Boghdady

**Affiliations:** 1 Department of Biochemistry, Faculty of Pharmacy, Cairo University, 23 Kasr El-Aini St., Cairo, 11562 Egypt; 2 Department of Pharmacology and Therapeutics, Faculty of Pharmacy, Pharos University in Alexandria (PUA), Canal El Mahmoudia Street, beside the Green Plaza Complex, Alexandria, 21648 Egypt; 3 Department of Biochemistry, Medical Research Institute, Alexandria University, 165 Al-Horreya Avenue, Hadara, Alexandria, 21561 Egypt

**Keywords:** Hepatocellular carcinoma, Astaxanthin, Doxorubicin, Wnt/β-catenin, Rat model, Biochemistry, Cancer, Cell biology, Drug discovery, Molecular biology, Oncology

## Abstract

Astaxanthin (ATX) is a potent natural antioxidant that shows promise against hepatocellular carcinoma (HCC). We investigated the therapeutic potential of ATX and its impact on Wnt/β-catenin pathway in a rat model, inducing HCC by nitrosodiethylamine (DEN) and carbon tetrachloride (CCl_4_). Rats were divided into five groups; I: control, II: HCC, III: HCC rats received ATX (5 days/week), IV: HCC rats received doxorubicin (DOX) weekly, V: HCC rats received combination therapy, for 4 weeks. Our results indicate that combination therapy significantly improved serum biomarkers, reducing alanine aminotransferase (ALT), aspartate aminotransferase (AST), and alpha-fetoprotein (AFP) levels by 45%, 35%, 49%, respectively compared to untreated HCC rats. Histopathological examination revealed the absence of hepato-carcinogenic nodules in the DOX- and combination-treated groups. Furthermore, combination therapy downregulated Wnt/β-catenin pathway components, decreasing frizzled-7 (FZD-7) by 56%, low-density lipoprotein receptor-related proteins 5/6 (LRP5/6) by 62%, β-catenin by 58%, and upregulating glycogen synthase kinase-3β (GSK3β) by 41% compared to untreated HCC rats. Consequently, the expression of key proteins such as cyclin D1 was ameliorated by 46%. While DOX monotherapy upregulated multidrug resistance protein-1 (MDR1) by 65%, combination therapy mitigated this increase by 36%. These findings highlight that ATX has potential therapeutic benefits in HCC treatment in rats.

**Supplementary Information:**

The online version contains supplementary material available at 10.1038/s41598-026-45680-1.

## Introduction

Hepatocellular carcinoma (HCC) remains a major global health burden, ranking as the sixth most prevalent cancer and the third leading cause of cancer-related mortality worldwide^1^. Egypt exhibits a particularly high incidence of HCC, ranking third in Africa and 15th globally^2^. Doxorubicin (DOX), an anthracycline antibiotic, is commonly used in HCC management due to its significant therapeutic potential; however, cardiotoxicity and drug resistance remain as major limitations^3^. Its mechanism of action involves inhibiting topoisomerase II, disrupting deoxyribonucleic acid (DNA) replication, and generating free radicals, thereby inducing cellular damage^4^. Due to limitations of the conventional chemotherapeutic agents, there is increasing interest in investigating alternative therapies to enhance chemotherapy efficacy and mitigate toxicity^5^. For instance, Licochalcone B has been shown to ameliorate liver cancer by targeting apoptotic genes and DNA repair systems^6^, while natural agents like camel milk and bee honey have demonstrated efficacy in regulating profibrotic cytokines in carbon tetrachloride (CCl_4_)-induced cirrhosis^7^. These studies underscore the validity of exploring natural antioxidants.

A key pathway of interest is the canonical Wnt/β-catenin pathway, which is essential in hepatic development, normal liver function, and tissue homeostasis; its dysregulation is implicated in various liver diseases and cancer^8^. Under normal conditions, in the absence of a Wnt ligand, cytoplasmic β-catenin levels are kept low through phosphorylation and destruction by the destruction complex, resulting in the repression of Wnt target genes^9^. Conversely, activation of Wnt signaling pathway via binding of Wnt ligands to their receptors disrupts the destruction complex. This leads to cytoplasmic β-catenin accumulation, and its translocation to the nucleus, where it functions as a transcription activator of target genes essential for cell proliferation and survival, such as cyclin D1, cellular myelocytomatosis (c-Myc) and multidrug resistance protein-1 (MDR1)^10^.

Astaxanthin (ATX), a xanthophyll carotenoid and red fat-soluble pigment, exhibits potent biological antioxidant activity compared to other carotenoids. It is naturally present in various marine organisms and microorganisms, such as shrimp, krill, salmon and *Haematococcus pluvialis (H. pluvialis)*, a green microalga rich in ATX content^11^. Its chemical structure comprises a six-membered ring with two terminal rings joined by conjugated double bonds, which not only determine its color but also govern its biological function^12^. ATX exhibits a diverse range of biological activities, including potent antioxidant activity attributed to its distinctive molecular structure, containing hydroxyl and keto moieties on each ionone ring^13^. It also possesses anti-lipid peroxidation activity, protecting the cell membrane by overlaying its terminal polar groups with the polar regions of the cell membrane, while fitting its strongly hydrophobic conjugated polyene structure to the inner non‑polar region. Thus, ATX spans biological membranes, maintaining the membrane structure, inhibiting lipid peroxidation, and acting as an antioxidant^14^.

Additionally, ATX demonstrates anticancer properties through various mechanisms, such as anti-proliferation, modulation of apoptosis, anti-oxidation, cell cycle arrest, and cell growth inhibition^15,16^. ATX has been shown to increase the levels of glycogen synthase kinase-3β (GSK3β) and suppress the nuclear transfer of β-catenin. It also modulates protein kinase B (Akt) and extracellular signal-regulated kinase (ERK) phosphorylation, consequently inhibiting the nuclear factor kappa B (NF-κB) and Wnt/β-catenin pathways^17^. Therefore, this study aims to investigate the possible therapeutic and/or adjuvant effects of ATX in rat model of HCC.

## Results

### Expression of β-catenin, GSK3β and AFP levels

**Fig. 1:**
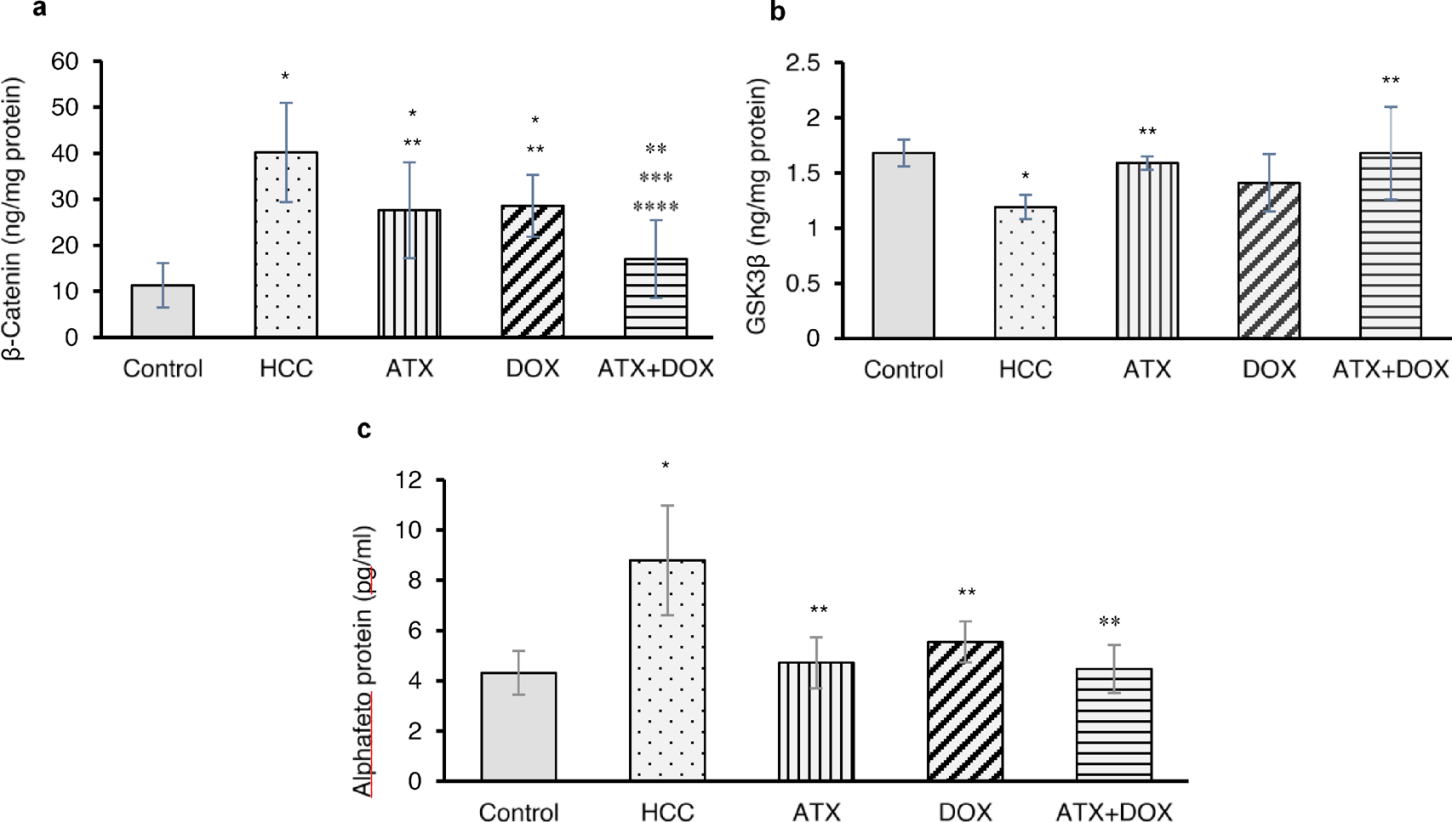
Effect of ATX and DOX on hepatic Wnt pathway proteins and AFP levels. Protein levels of (**a**) β-catenin, (**b**) GSK3β, and (**c**) AFP were assessed using ELISA technique. Data are expressed as mean ± standard deviation (SD) (*N* = 6). Statistical analysis was performed using one-way ANOVA followed by Tukey’s post-hoc test, with significance set at (*P* < 0.05). * Statistically significant with control group, ** Statistically significant with HCC group, *** Statistically significant with ATX-treated group, and **** Statistically significant with DOX-treated group.

Significant alterations in β-catenin expression levels were observed among the experimental groups. Specifically, untreated HCC rats exhibited a considerable elevation in β-catenin expression, showing an approximately 3.5-fold increase compared to the control group (Fig. 1a). Conversely, rats treated with ATX, DOX, or combination therapy showed reductions in β-catenin expression levels by 31%, 29%, and 58%, respectively, relative to the untreated HCC group. Moreover, HCC rats treated with combination therapy exhibited a notably decreased expression of β-catenin compared to ATX- and DOX-treated rats by approximately 38%, and 40%, respectively.

Regarding GSK3β expression, untreated HCC rats exhibited decreased levels of GSK3β protein compared to the control group (0.71-fold decrease). However, HCC rats treated with either ATX or combination therapy showed a remarkable increase in GSK3β levels compared to untreated rats, by approximately 33.6% and 41%, respectively (Fig. 1b).

Additionally, alpha-fetoprotein (AFP), a glycoprotein serving as a key biomarker for HCC diagnosis and monitoring, demonstrated elevated levels in untreated HCC rats compared to the control group, reaching a 2-fold increase (Fig. 1c). Conversely, HCC rats treated with either ATX or DOX exhibited significant reduction in AFP level, decreasing by approximately 46% and 37%, respectively, compared to untreated rats. HCC rats treated with combination therapy showed a remarkable decrease in AFP levels, reducing them by 49% compared to the untreated group.

### Expression of FZD-7 and LRP5/6 receptors by western blot

**Fig. 2:**
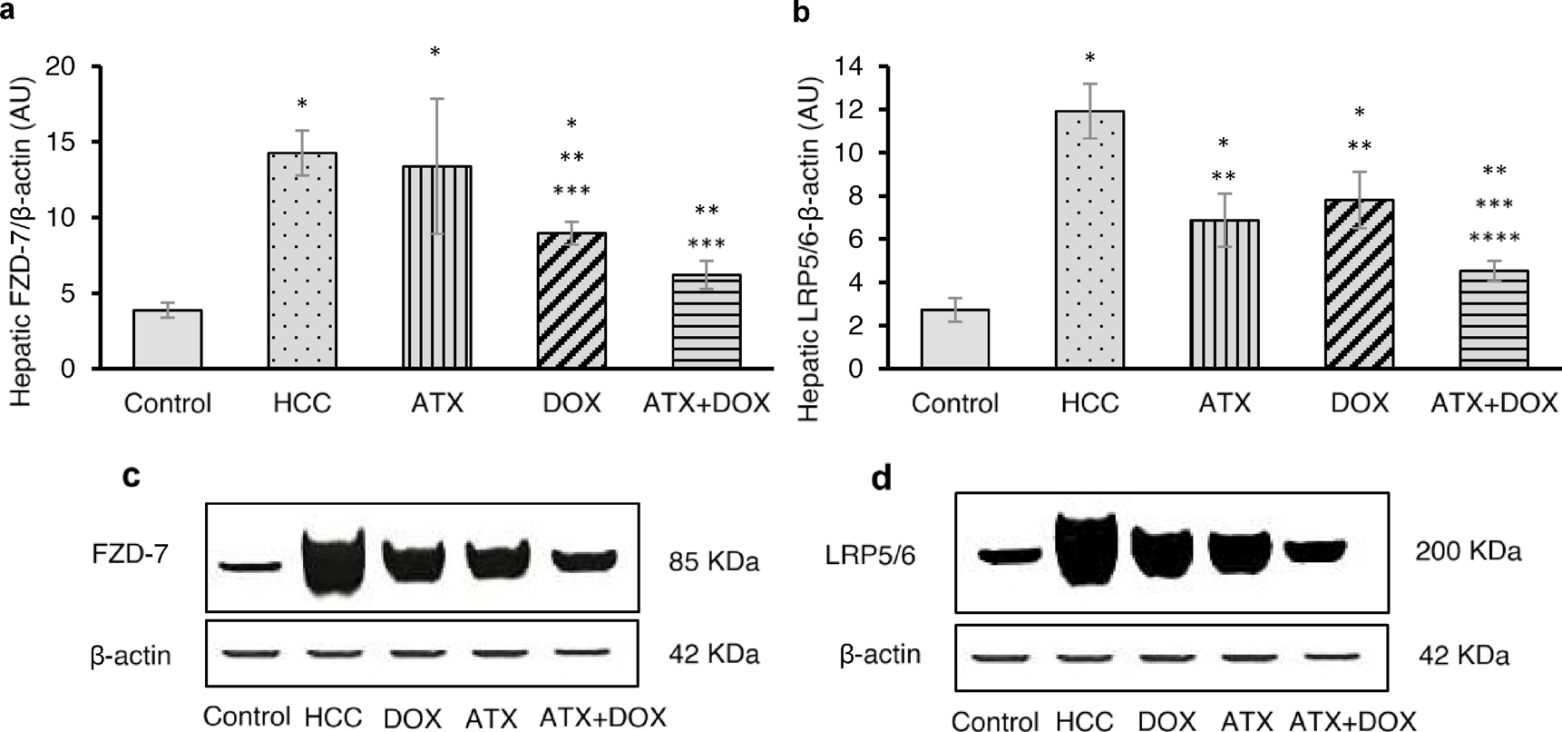
Effect of ATX and DOX on FZD-7 and LRP5/6 receptor expression. Protein expression was analyzed by western blot. The bar graphs represent the quantitative analysis of (**a**) FZD-7 receptor and (**b**) LRP5/6 receptor. (**c**) Representative western blot bands for FZD-7, and (**d**) representative western blot bands for LRP5/6. β-actin was used as a loading control. All lanes were run on the same gel and detected in a single exposure. Membranes were not cut prior to hybridization, and images are cropped for clarity. Original, uncropped images of the western blots are presented in Supplementary Figures S1–S9. Data are expressed as mean ± standard deviation (SD) (*N* = 3). Statistical analysis was performed using one-way ANOVA followed by Tukey’s post-hoc test, with significance set at (*P* < 0.05). * Statistically significant with control group, ** Statistically significant with HCC group, *** Statistically significant with ATX-treated group, and **** Statistically significant with DOX-treated group.

Frizzled-7 (FZD-7) receptor expression levels were significantly elevated in untreated HCC rats compared to the control group, showing an approximately 3.7-fold increase. (Figure 2a and c). Conversely, HCC rats treated with either DOX or combination therapy displayed notable expression reductions of approximately 37% and 56%, respectively, compared to the untreated group. Moreover, they also exhibited a decrease in FZD-7 receptor expression by 33% and 54%, respectively, compared to those treated with ATX alone.

Similarly, low-density lipoprotein receptor-related proteins 5/6 (LRP5/6) receptor expression levels were notably increased in untreated HCC rats compared to the control group, showing a significant 4.4-fold increase (Fig. 2b and d). Treatment with either ATX, DOX, or combination therapy resulted in decreased expression of LRP5/6 by approximately 42%, 34%, and 62%, respectively, relative to untreated rats. HCC rats treated with combination therapy showed a notable decrease in LRP5/6 receptor expression, reducing levels by 34% compared to those treated with ATX alone, and by 42% compared to those treated with DOX alone.

### Gene expression of cyclin D1, c-Myc, and MDR1 by quantitative real-time polymerase chain reaction (qRT-PCR)

**Fig. 3:**
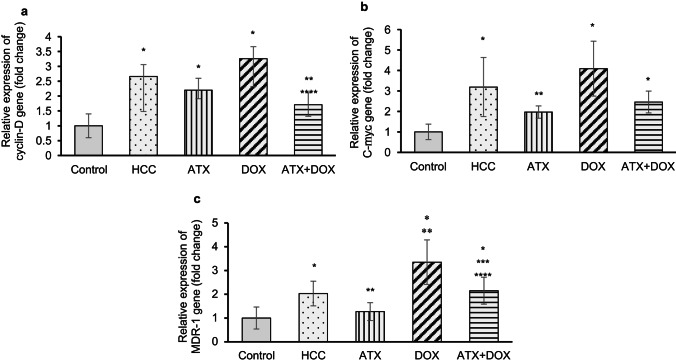
Impact of ATX and DOX on the gene expression of Wnt target genes. Relative messenger ribonucleic acid (mRNA) expression levels of (**a**) cyclin D1, (**b**) c-Myc, and (**c**) MDR1 were quantified using qRT-PCR. Data are expressed as mean ± standard deviation (SD) (*N* = 6). Statistical analysis was performed using one-way ANOVA followed by Tukey’s post-hoc test, with significance set at (*P* < 0.05). * Statistically significant with control group, ** Statistically significant with HCC group, *** Statistically significant with ATX-treated group, and **** Statistically significant with DOX-treated group.

The cyclin D1 gene, an important regulator of the cell cycle and a direct target of Wnt/β-catenin pathway, showed significant upregulation in the untreated HCC group, indicating increased cell cycle progression. Untreated HCC rats exhibited an approximately 3.1-fold increase in gene expression compared to control rats (Fig. 3a). In contrast, rats treated with either ATX or combination therapy showed downregulation in gene expression compared to untreated HCC rats by approximately 30% and 46%, respectively.

It is important to note that c-Myc, a proto-oncogene and target of Wnt/β-catenin pathway, promotes cellular proliferation, growth, and survival, thus contributing to oncogenesis. Untreated HCC rats displayed significant upregulation in c-Myc expression compared to the control group, with an approximately 3.8-fold increase (Fig. 3b). On the other hand, HCC rats treated with ATX exhibited significant downregulation of c-Myc expression by about 48% relative to the untreated group.

The MDR1 gene, also a target of the Wnt/β-catenin pathway, plays a crucial role in HCC by contributing to chemoresistance, thereby worsening treatment efficacy and patient outcomes. Untreated HCC rats exhibited significant upregulation of MDR1 expression compared to the control group (approximately 2-fold increase) (Fig. 3c). Whereas HCC rats treated with ATX alone exhibited significant downregulation of gene expression by about 37% relative to the untreated group, those treated with DOX displayed notable upregulation by approximately 65%. However, rats treated with either ATX or combination therapy displayed significantly lower levels of MDR1 expression compared to those treated with DOX alone (reductions of approximately 62% and 36%, respectively). Furthermore, rats treated with ATX exhibited significantly lower MDR1 expression (by approximately 69%) compared to the combination group, while the DOX-treated group displayed significant upregulation compared to the combination group (by about 36%).

### Hepatic glutathione and MDA levels

**Fig. 4:**
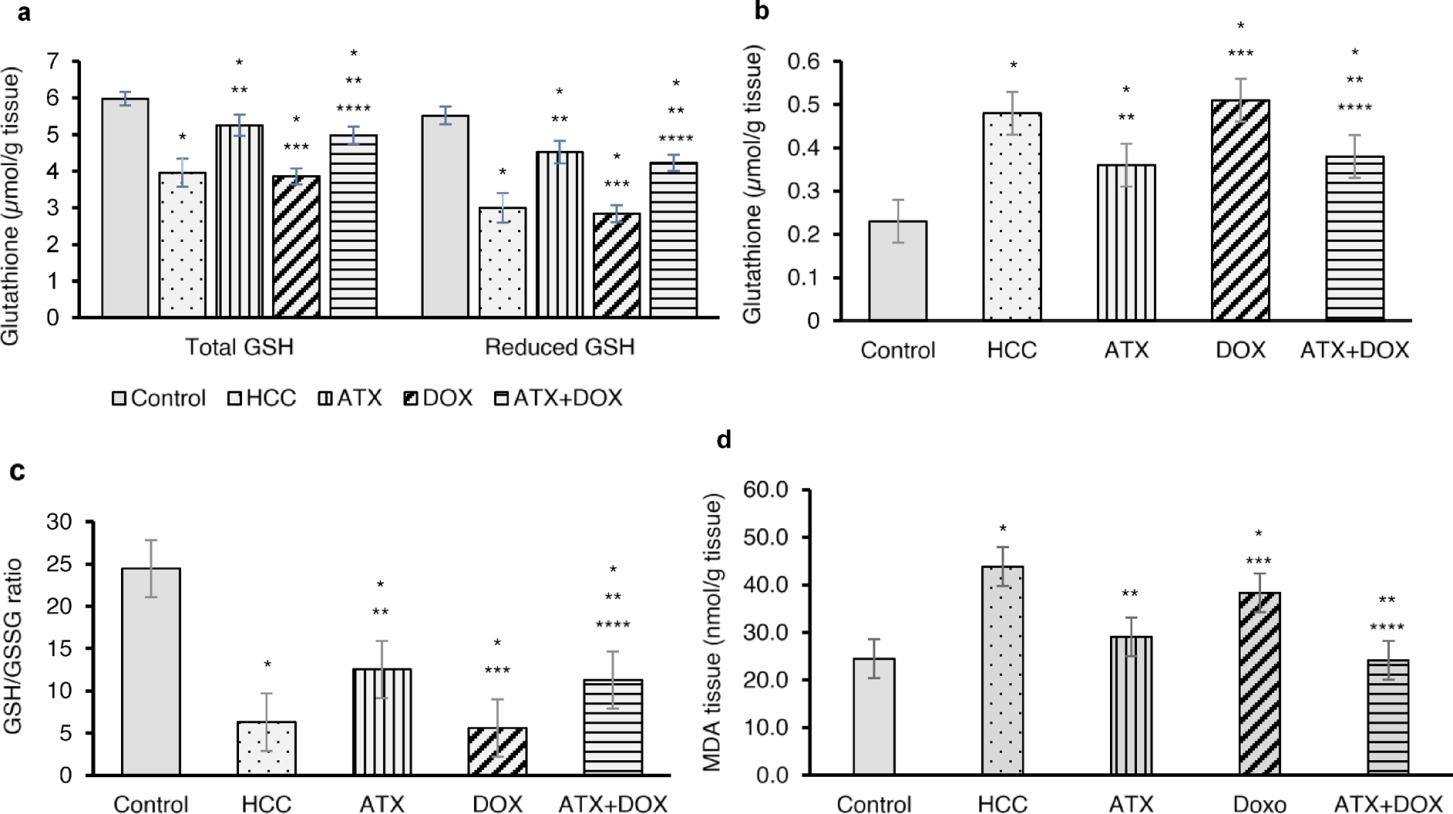
Effect of ATX and DOX on hepatic oxidative stress markers. Assessment of (**a**) total and reduced glutathione, (**b**) oxidized glutathione, (**c**) GSH/GSSG ratio, and (**d**) MDA levels in liver tissue. Data are expressed as mean ± standard deviation (SD) (*N* = 6). Statistical analysis was performed using one-way ANOVA followed by Tukey’s post-hoc test, with significance set at (*P* < 0.05). * Statistically significant with control group, ** Statistically significant with HCC group, *** Statistically significant with ATX-treated group, and **** Statistically significant with DOX-treated group.

The hepatic glutathione system, a critical antioxidant mechanism, plays a vital role in maintaining detoxification processes and cellular redox homeostasis. In HCC, this system undergoes dysregulation, altering the oxidative stress response. Untreated HCC rats exhibited a significant decrease in total and reduced glutathione (GSH) levels (0.66- and 0.54-fold decrease, respectively) and an increase in oxidized glutathione (GSSG) levels (approximately 2-fold increase) compared to the control group (Fig. 4a and b). However, treatment with either ATX or combination therapy resulted in significantly higher levels of total and reduced GSH compared to the untreated group (increases of approximately 33%, 26%, 51%, and 41%, respectively). These treatments also resulted in significantly lower levels of GSSG, reducing them by approximately 25% and 21%, respectively.

Additionally, untreated HCC rats exhibited a significantly decreased GSH/GSSG ratio (0.26-fold decrease) compared to the control group. Conversely, treatment with either ATX or combination therapy resulted in significantly higher GSH/GSSG ratios compared to the untreated group (increases of approximately 99% and 79%, respectively). These groups also demonstrated higher ratios compared to the DOX-treated group, by about 124% and 101%, respectively (Fig. 4c).

Tissue malondialdehyde (MDA) levels are considered important biomarkers reflecting the oxidative stress levels and lipid peroxidation. Untreated HCC rats demonstrated notably elevated tissue MDA levels compared to the control group, reaching a 1.8-fold increase (Fig. 4d). However, HCC rats treated with ATX, combination therapy, or DOX exhibited reductions in tissue MDA levels by approximately 34%, 45%, and 13%, respectively, in comparison to the untreated group. Furthermore, HCC rats treated with either ATX or combination therapy showed significantly lower tissue MDA levels (by approximately 24% and 37%, respectively) compared to the DOX-treated group. Notably, ATX and combination therapy restored tissue MDA to normal levels in HCC rats.

### Hepatic AST and ALT activities

**Fig. 5:**
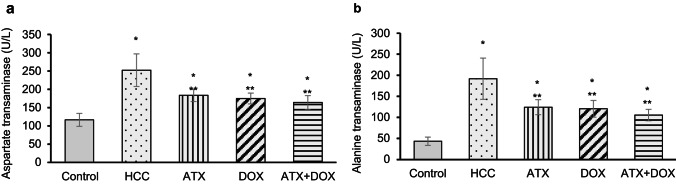
Evaluation of liver function enzymes. Serum activities (**a**) AST and (**b**) ALT. Data are expressed as mean ± standard deviation (SD) (*N* = 6). Statistical analysis was performed using one-way ANOVA followed by Tukey’s post-hoc test, with significance set at (*P* < 0.05). * Statistically significant with control group, ** Statistically significant with HCC group.

Hepatic aspartate aminotransferase (AST) and alanine aminotransferase (ALT) are key biomarkers for assessing the liver function, revealing the extent of liver injury and damage in HCC. Untreated HCC rats exhibited notably higher serum AST and ALT activity (approximately 2.2- and 4.4-fold increases, respectively) compared to control rats (Fig. 5a and b). On the other hand, HCC rats treated with ATX, DOX, or combination therapy showed significantly reduced serum AST activity compared to untreated rats (reductions of approximately 27%, 31%, and 35%, respectively) and reduced serum ALT activity (reductions of approximately 35%, 37%, and 45%, respectively). However, AST and ALT activities in groups treated with either ATX or combination therapy remained significantly higher than control values.

### Histopathological architecture of liver tissue

**Fig. 6:**
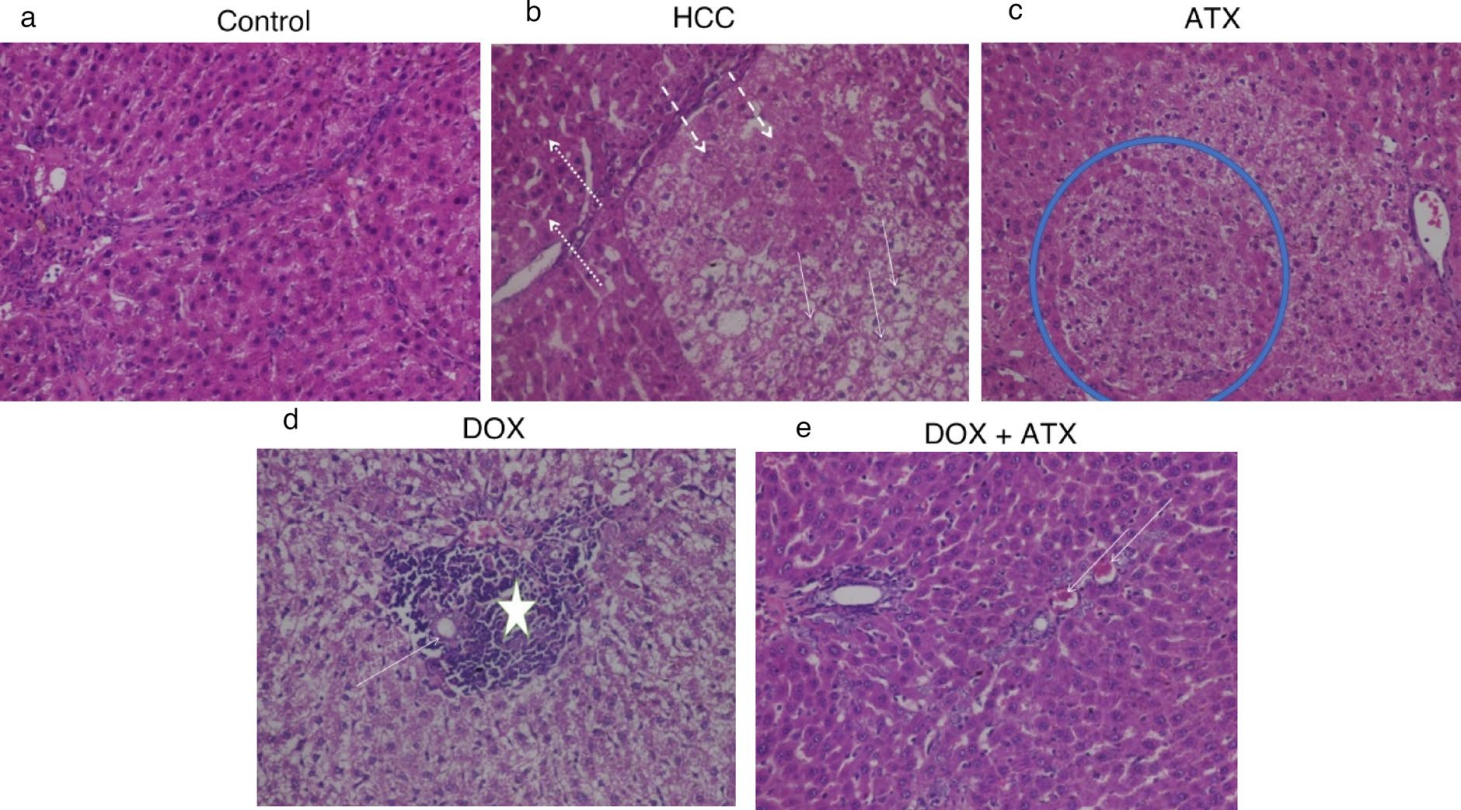
Histopathological examination of liver tissue sections. Representative photomicrographs of liver sections stained with hematoxylin and eosin (H&E, X400): (**a**) control group showing normal hepatic architecture, (**b**) untreated HCC group displaying hepato-carcinogenic nodules and hydropic degeneration, (**c**) ATX-treated group, (**d**) DOX-treated group, and (**e**) combination treatment group showing absence of nodules and mild congestion. All treatments were given for 4 weeks, and histological assessment was conducted in duplicates (*N* = 2).

In the liver sections of control rats, normal hepatic architecture was observed, characterized by hexagonal hepatic lobes, intact portal triads, and central veins (Fig. 6a). In contrast, untreated HCC rats exhibited hepato-carcinogenic nodules, with hepatocytes showing varied characteristics including highly eosinophilic and finely granular cytoplasm, as well as a high degree of hydropic degeneration. Nuclear hyperchromasia was also noted (Fig. 6b).

Treatment with ATX alone revealed significant abnormalities, including well-demarcated henodules of abnormal hepatocytes without the development of fibrous capsules (Fig. 6c). In DOX-treated rats, hepato-carcinogenic nodules were absent, but were replaced by large foci of necrotized hepatocytes. Aberrant arterioles were observed within these foci, and adjacent hepatocytes displayed marked hydropic degeneration with vesicular nuclei (Fig. 6d). Additionally, rats treated with a combination of ATX and DOX showed an absence of hepato-carcinogenic nodules; instead, mild dilation of hepatic sinusoids and congestion of hepatic vasculature were observed (Fig. 6e).

## Discussion

The current investigation aimed to explore the molecular impact of ATX on chemically induced HCC in rats, specifically focusing on its ability to potentiate the efficacy of conventional HCC drugs, such as DOX. Our results indicate that ATX acts via a multi-targeted mechanism: it suppresses the canonical Wnt/β-catenin pathway parameters, while restoring the tumor suppressor GSK3β. It also downregulated the expression of oncogenes associated with the pathway and HCC progression, such as cyclin D1, c-Myc, and the chemoresistance gene MDR1. Furthermore, ATX administration correlated with improved hepatic function and antioxidant status, suggesting its value as a protective adjuvant.

Unlike xenograft models often used in similar studies^18,19^, we utilized a nitrosodiethylamine (DEN) and CCl_4_-induced model which mimics the inflammatory and fibrotic microenvironment of human HCC^20^. Histopathological examination revealed that hepatocytes of HCC rats showed adenocarcinoma, indicated by proliferated neoplastic ductal epithelium with cellular and nuclear pleomorphism. Similar histopathological changes were reported in Sengupta and EL Sayed^21,22^. These histological changes were associated with a loss of normal liver architecture and function, increasing hepatotoxicity and elevating serum ALT, AST activities, and AFP levels. This is interpreted as a result of the high toxicity of DEN+CCl_4_, which is metabolized in the liver, through bioactivation into reactive trichloromethyl radicals (CCl^·^_3_), which alkylate DNA and initiate chain reactions of lipid peroxidation in hepatocyte membranes causing liver degeneration^23,24^.

Treatment with ATX alone offered partial histological improvement, consistent with findings by Ohno^25^. In addition, treatment with DOX resulted in mildly ameliorated liver histology with moderate degenerative changes, necrotic regions with inflammatory cell infiltration, and severe collagen fiber deposition, consistence with Alansari & Eskandrani^26^. Recent studies on other natural antioxidants support these findings; for example, Sidr honey has been reported to abrogate oxidative stress and downregulate fibrogenic genes in similar thioacetamide-induced fibrosis models^27^. Notably, using ATX as an adjuvant treatment with DOX markedly improved hepatocytic damage, enhancing cellular integrity and reducing inflammation, necrosis, and fibrosis. This suggests the restoration of liver architecture in chemically induced HCC rats. Thus, integrating ATX with DOX in treatment regimens shows promising enhancement in the anticancer efficacy of conventional chemotherapy.

Improvements in serum parameters, such as ALT, AST, and AFP were also observed, with the most notable ameliorative effects seen in rats co-treated with DOX and ATX, followed by those treated with ATX alone, and finally those treated with DOX alone. These findings align with previous reports indicating that carotenoid extracts, such as ATX, reduce liver enzyme levels^28^, highlighting the potent antioxidant properties of ATX and its potential to mitigate liver damage^29,30^. This hepatoprotective mechanism parallels the action of melatonin, which has been found to mitigate hepatic fibrosis via antioxidant activity and the modulation of proinflammatory cytokines^31^.

The dysregulation of Wnt/β-catenin signaling is a hallmark of hepatocarcinogenesis^32^. β-catenin, a key downstream component, is essential for liver development, proliferation, and regeneration. The pathway is activated when Wnt ligands attach to specific cell surface receptors, such as FZD-7 and LRP5/6, leading to β-catenin stabilization and nuclear translocation. In the absence of Wnt signaling, the β-catenin is targeted for degradation by a destruction complex including adenomatous polyposis coli (APC), Axin, and GSK3β. This canonical Wnt/β-catenin signaling pathway is recognized as a key driver of tumorigenesis, particularly in HCC^33^.

Most research on natural products in HCC focuses on downstream targets (like cyclin D1 and c-Myc)^34^. Our study dissects the pathway upstream, providing novel data on the modulation of the FZD-7 and LRP5/6 co-receptor in a chemically induced rat model. We observed elevated hepatic expression of FZD-7 and LRP5/6 receptors, along with β-catenin accumulation and GSK3β inactivation in HCC rats, aligning with the findings of Tung^35^ and Preziosi^36^. Treatment with DOX or ATX partially restored the elevated expression levels of FZD-7 and LRP5/6 receptors, with the most significant effects noted in rats receiving combination therapy. Treatment with DOX led to decreased β-catenin levels, while treatment with ATX resulted in a more pronounced decrease. This decline may stem from the inhibition of the Wnt/β-catenin pathway via the blockade of GSK3β phosphorylation, resulting in reduced β-catenin nuclear translocation^17^. Consequently, the combination treatment group exhibited the lowest β-catenin levels.

GSK3β is a serine/threonine kinase that phosphorylates and inactivates proteins by targeting them for proteasomal degradation. It has two phosphorylation sites: it is activated by phosphorylation at Tyr-216 and inactivated by phosphorylation at Ser-9 and has been associated with many pathological conditions. GSK3β act as a tumor suppressor by suppressing the Wnt/β-catenin pathway through the phosphorylation of β-catenin, targeting it for ubiquitination and proteasomal degradation. When GSK3β is inactivated, it leads to de-phosphorylation of β-catenin, its stabilization, and translocation to the nucleus, resulting in the expression of the target genes and displaying oncogenic properties^37^.

We observed significant downregulation of GSK3β in untreated HCC rats, leading to β-catenin stabilization and subsequent activation of target genes promoting tumor growth. This decrease aligns with findings from Ban^38^. Our study revealed that treatment with ATX resulted in increased levels of GSK3β expression compared to the HCC group. This may be explained by the anticancer properties of ATX, which influence cellular signaling mechanisms relevant to tumor development. Since GSK3β activity is regulated by phosphorylation, ATX may reduce the phosphorylation of GSK3β, resulting in elevated GSK3β levels, as reported by Li^17^ and Kowshik^39^.

Additionally, we observed a notable increase in GSK3β expression in the combination-treated group compared to the HCC group, approaching normal levels. The combination treatment may synergistically affect GSK3β expression and activity, with DOX and ATX indirectly affecting GSK3β and influencing its phosphorylation and activity^40^. These modulations likely restored GSK3β to near-normal levels.

We posit that ATX exerts its inhibitory effect at two distinct levels of this pathway: (1) receptor level: the observed downregulation of LRP5/6 suggests ATX may interfere with the initial ligand-receptor signal transduction or receptor turnover. (2) destruction complex level: ATX treatment restored GSK3β protein levels. GSK3β inactivation (often via phosphorylation at Ser9) is a common event in cancer that prevents β-catenin degradation^33^. Our results suggest ATX may prevent this inhibitory phosphorylation, maintaining GSK3β in its active state, thereby facilitating the continuous degradation of β-catenin. This aligns with findings by Li et al.^17^, who demonstrated that carotenoids could modulate kinase activity to suppress oncogenic signaling.

However, it is important to acknowledge the limitation of the current study. While we observed a reduction in total β-catenin protein and a downregulation of its downstream target genes (cyclin D1, c-Myc), which serves as a proxy for reduced transcriptional activity, we did not provide direct functional evidence regarding β-catenin localization. As the translocation of β-catenin from the cytoplasm to the nucleus is the hallmark of pathway activation. Future studies utilizing nuclear/cytoplasmic fractionation or immunohistochemical staining are recommended to definitively confirm the blockade of nuclear translocation by ATX in this HCC model^41^.

Activation of the Wnt pathway in HCC triggers various oncogenic processes, promoting cell proliferation by upregulating target genes like cyclin D1, driving uncontrolled cell division and tumor progression. In our investigation, HCC rats exhibited elevated cyclin D1 levels compared to control rats, whereas those treated with ATX alone or in combination with DOX displayed reduced cyclin D1 expression, approaching normal levels. Previous research has shown that ATX decreases cyclin D1 expression, inducing cell cycle arrest in DEN-obese mice^25^. Although the precise mechanisms of ATX remain incompletely elucidated, it has been observed to inhibit NF-κB and Wnt pathways, leading to mitochondrial-mediated apoptosis and downregulation of proliferation-related genes, such as cyclin D1^42^. DOX, acts as a chemotherapeutic agent inducing DNA damage and stress in cancer cells; consequently, cancer cells may upregulate cyclin D1 expression to counteract this stress and facilitate survival and proliferation, which could explain the observed elevated cyclin D1 expression in DOX-treated rats^43,44^.

We also observed an upregulation of c-Myc mRNA gene expression in HCC rats, consistent with previous findings that DEN-induced HCC leads to increased c-Myc expression^45^. Treatment with ATX alone led to downregulation of gene expression, approaching normal levels. Similarly, rats treated with combination therapy exhibited downregulation of gene expression compared to untreated rats and those treated with DOX alone, potentially due to the effects of ATX. Overexpression of c-Myc is a hallmark of many cancers, including HCC. DOX-induced stress can lead to upregulation of survival pathways mediated by c-Myc as a compensatory mechanism; thus, upregulation of c-Myc expression may reflect an adaptive response to mitigate the cytotoxic effects DOX^46,47^.

Additionally, elevated expression of the MDR1 gene was observed in HCC rats. This corroborates previous research by Kuo^48^, who attributed this overexpression to various inducers, including chemical induction of HCC by DEN, which promotes carcinogenesis by upregulating MDR1 expression and activating the NF-κB pathway.

While previous studies have established ATX as a potent antioxidant^49^, our study is among the first to provide in-vivo evidence that ATX functions as a chemosensitizer for DOX specifically by targeting the Wnt-mediated expression of MDR1. Addressing the challenge of DOX resistance in HCC treatment, our investigation revealed that rats treated with DOX exhibited higher expression of the MDR1 gene compared to untreated rats. This paradoxical upregulation represents a well-documented mechanism of acquired chemoresistance. Exposure to DOX induces cellular stress which triggers an adaptive response, leading to the overexpression of ATP-binding cassette (ABC) transporters such as P-glycoprotein (MDR1)^50^. This efflux pump actively transports DOX out of the tumor cells, reducing its intracellular accumulation and therapeutic efficacy^51^. Crucially, our data demonstrated that treatment with ATX alone counteracts this resistance mechanism and showed notable downregulation of MDR1 expression, approaching normal levels. Furthermore, rats receiving combination therapy also exhibited reduced gene expression compared to those treated with DOX alone. Since MDR1 is a direct transcriptional target of Wnt/β-catenin pathway, the ability of ATX to inhibit β-catenin nuclear accumulation likely prevents the DOX-induced upregulation of MDR1. This indicates the protective effects of ATX, preserving the intracellular retention of DOX and enhancing its cytotoxic potential against HCC cells^52^.

ATX exhibits robust antioxidative properties that can scavenge free radicals and mitigate oxidative stress, as reported by Islam^53^. The antioxidant efficacy of ATX is attributed to the unique chemical structure of ATX. Unlike β-carotene, ATX possesses polar hydroxyl and keto groups on its ionone rings, allowing it to span the entire transmembrane bilayer, with both polar ends of the molecule interacting with the polar zones of the bilayer while the remaining portion aligns with the hydrocarbon chain. This orientation effectively and securely anchors the membrane, stabilizing it against the oxidative assault of chemotherapy and carcinogens^54^.

Furthermore, hepatoprotective effects of ATX are exerted not only by scavenging free radicals and reducing lipid peroxidation but also by modulating the nuclear factor erythroid 2–related factor 2 (Nrf2) pathway. This pathway serves as a master transcriptional regulator of cellular antioxidants, orchestrating the expression of hepatic antioxidant enzymes^55^. Consequently, ATX supplementation triggers the antioxidant defense system, enhancing cellular resilience against oxidative damage.

Consistent with established literature regarding hepatic oxidative injury, our study demonstrated elevated MDA levels in the hepatic tissue of HCC rats^56,57^. This was accompanied by a significant disruption in cellular redox homeostasis, characterized by a marked reduction in both total and reduced GSH, alongside an elevation in GSSG. Consequently, a severe suppression of the GSH/GSSG ratio was evident in the DEN/CCl_4_-induced model. These observations mirror clinical findings where HCC patients exhibit depleted GSH levels^58–60^ and diminished glutathione peroxidase (GPx) activity^61,62^ relative to healthy controls or hepatitis patients.

Regarding therapeutic intervention, DOX monotherapy failed to ameliorate this oxidative burden or improve the GSH system. In contrast, ATX treatment alone markedly attenuated these disturbances. Notably, the co-administration of DOX and ATX yielded a synergistic effect, enhancing the anti-HCC efficacy of DOX and completely normalizing hepatic MDA levels.

Crucially, this restoration of redox balance has mechanistic implications beyond simple cytoprotection, as oxidative stress is a driver of HCC progression rather than merely a symptom. Reactive oxygen species (ROS) have been shown to aberrantly activate the Wnt signaling pathway, even in the absence of Wnt ligands, by oxidizing nucleoredoxin and releasing the signaling protein Dishevelled (Dvl)^63^. In our study, ATX acted as a potent scavenger, reducing ROS, lowering MDA, and restoring the GSH/GSSG ratio. By neutralizing the ROS required to sustain this signaling axis, ATX provides a secondary, indirect mechanism for suppressing the Wnt pathway.

## Materials and methods

### Experimental animals

Adult male Wistar albino rats weighing approximately (140 ± 20 g) were procured from the animal house of Pharos University, Alexandria, Egypt. The animals were housed in groups of five per cage under strictly controlled laboratory conditions, including humidity of (60 ± 10%), room temperature maintained at (25 ± 2 °C) and 12/12-h light/dark cycle. They all had unrestricted access to food and water, and constant environmental conditions were maintained before and after the experimental period. Humane sacrifice procedures and light anesthesia were used to guarantee that animals did not suffer during the experiment. Humane endpoints were defined to prevent unnecessary suffering. Animals were monitored daily for signs of distress, including significant weight loss, inability to access food or water, severe lethargy, or ruffled fur. Any animal reaching these endpoints was immediately euthanized via cervical dislocation under thiopental sodium anesthesia^64^.

### Ethics approval

The research study was performed in accordance with the outlined guidelines in the National Institutes of Health’s Guide for the Care and Use of Laboratory Animals (NIH Publication No. 85-z23, revised 2011). All experiments conformed to the ARRIVE guidelines (https://arriveguidelines.org)^65^ and ethical approval was received from the Ethics Committee of Faculty of Pharmacy, Cairo University, Egypt. (Permit Number: BC 2581) to ensure compliance with established standards for humane animal research.

### Drugs

DEN was supplied from Sigma-Aldrich Chemical Co., St. Louis, USA (Cat. No. N0258) and dissolved in normal saline (0.9% NaCl), CCl_4_ was received from Alpha Chemika, Mumbai, India (Cat. No. AL1144), DOX was obtained from Hikma Specialized Pharmaceuticals, Cairo, Egypt and ATX from Carbosynth Ltd. Berkshire, UK (Cat. No. FA18001).

### Induction of HCC

HCC was induced by administering a single intraperitoneal injection of DEN dissolved in normal saline at a dose of 200 mg/Kg. After 2 weeks, rats received subcutaneous injection of CCl_4_ dissolved in olive oil at a dose of 3 ml/Kg, administered once weekly for 6 consecutive weeks to enhance the carcinogenic effect of DEN^66^.

### Experimental design

Fifty rats were assigned into five groups randomly to ensure unbiased allocation, each consisting of 10 rats. Group I: control rats received normal saline and olive oil as a vehicle. Group II: HCC rats. Group III: HCC rats were treated with ATX dissolved in olive oil (25 mg/kg via oral gavage) for 5 days/week^67^. Group IV: HCC rats were treated with DOX dissolved in normal saline (2 mg/kg/weekly; i.p)^68^. Group V: HCC rats received a combination of ATX (25 mg/kg by oral gavage for 5 days/week) and DOX (2 mg/kg weekly; i.p), all treatments were given for 4 weeks. To minimize observer bias, the investigators performing the biochemical and histopathological assessments were blinded to the group allocation during the data acquisition and analysis phases.

### Blood samples and tissue processing

After the treatment period, rats were sacrificed by cervical dislocation under light anesthesia by thiopental sodium as described by El-Naggar^69^. Blood samples were taken from the retro-orbital area and allowed to clot for 20 min before being centrifuged at 2000×g for 10 min to separate the serum. The collected serum was divided into aliquots and stored at − 80 °C for subsequent analysis of AFP, AST, and ALT. The liver was then carefully removed, rinsed with saline and dried. A part of the liver was fixed in 10% formalin for histopathological examination, while another part was used for determination of MDA and GSH levels, β-catenin and GSK3β levels by enzyme-linked immunosorbent assay (ELISA), FZD-7 receptor and LRP5/6 coreceptors by western blot, cyclin D1, c-Myc, and MDR1 expression levels by qRT-PCR.

### Serum AST and ALT activities

The serum activities of AST and ALT were assayed. AST activity was measured by the provided kit (Spectrum Diagnostics, Egypt, Cat. No. AST: 260001) according to the manufacturer’s instructions, while ALT activity was measured by the commercially available kit (Spectrum Diagnostics, Egypt, Cat. No. ALT: 265002) following the manufacturer’s protocol.

### Hepatic GSH and MDA levels

Determination of total and GSSG was employed as described by Griffith^70^. The rate of formation of 5-thio-2-nitrobenzoic acid (TNB), indicative of GSH oxidation, was monitored by recording the change in the absorbance at 412 nm per minute (∆A/min). GSH levels expressed as µmol/g tissue. Also, MDA levels in rats’ liver tissue homogenate were assessed using thiobarbituric acid (TBA) assay as described by Draper & Hadley^71^. In the presence of heat, MDA, the end product of the hepatic lipid peroxidation reacts with TBA to produce a pink chromogen. The intensity of the color, reflecting MDA concentration, was measured at 532 nm. MDA level was expressed as nmol/g tissue.

### Enzyme-linked immunosorbent assay

β-catenin and GSK3β levels were determined in liver tissue homogenate according to the manufacturer’s instructions using rat-specific ELISA kits; β-catenin (Cloud-Clone Corp, USA, Cat. No. SEB012Ra) and GSK3β (Abcam, UK, Cat. No. ab123454). Also, serum AFP level was determined by rat-specific ELISA kit (Chongqing Biospes Co., Ltd, Cat. No. BAYEK 2963), adhering to the manufacturer’s instructions. Protein content for tissue homogenate was determined using Lowry method^72^ and results expressed as ng/mg protein for β-catenin, ng/mg protein for GSK3β and pg/ml for AFP.

### Western blot analysis

Western immunoblotting was carried out as described by Harlow and Lane^73^. Total protein was isolated using TriFast reagent (Peqlab, VWR, USA) via a tri-phasic extraction. Liver tissues were homogenized in 1 mL TriFast, and proteins were precipitated from the organic phase with isopropanol and following three washes in 0.3 M guanidinium hydrochloride in 95% ethanol, pellets were solubilized in 1% sodium dodecyl sulfate (SDS). Protein concentration was determined by Bradford assay at 595 nm using a bovine serum albumin (BSA) standard curve. For western blotting, 30 µg of protein was resolved on 10% SDS-polyacrylamide gel electrophoresis (SDS-PAGE) and electro-transferred to Amersham™ Hybond^®^ P membranes, polyvinylidene fluoride (PVDF) (Merck, UK, CAT. No. GE10600023). Membranes were blocked for 1 h in 5% non-fat dry milk in tris-buffered saline with Tween 20 (TBST) (25 mM Tris, 0.15 M NaCl, 0.1% Tween 20) and incubated overnight at 4 °C with primary antibodies, purchased from Abcam, USA, for FZD-7 (1 µg/mL dilution, Cat. No. ab64636), Cell Signaling Technology, USA, for LRP5/6 (1:1000 dilution, Cat. No. #5440), and Abcam, USA, for β-actin (1 µg/mL dilution, Cat. No. mAbcam 8226). After washing, membranes were incubated with HRP-conjugated secondary antibody (Abcam, USA, Cat. No. ab6721) (0.1–0.5 µg/mL) for 1 h at room temperature. β-actin was used as a loading control. Immunoreactive bands were captured using a gel documentation system (GelDoc-it, UVP, UK) and quantified via densitometry using TotalLab analysis software (Ver. 1.0.1). Results were expressed as arbitrary units (AU) after normalization with β-actin protein expression. Full-length, uncropped images of the western blots, including molecular weight markers and membrane edges, are provided in the Supplementary Information file.

### Quantitative RT-PCR analysis

Quantitative analysis of cyclin D1, c-Myc, and MDR1 genes expression in liver tissues were done using qRT-PCR. Initially the isolated RNA was reverse transcribed into complementary DNA (cDNA) using the reverse transcriptase enzyme. This cDNA was then amplified using specific primers by PCR (Table 1).

Total RNA was extracted from liver tissues using miRNeasy Mini Kit (Qiagen, USA, Cat. No. 217004) according to the manufacturer’s instructions. Subsequently, the extracted RNA was reverse transcribed using viva cDNA synthesis kit (Vivantis, Malaysia, Cat. No. cDSK01-050) following the manufacturer’s instructions. The obtained cDNA was utilized for qRT-PCR using ViPrimePLUS *Taq* qPCR Green Master Mix I (Vivantis Technologies, Selangor Darul Ehsan, Malaysia; Cat. No. QLMM12). The reactions were performed with CFX Connect Real-Time System (Bio-Rad, USA) device using specific primer sequences for the genes of interest and normalized to the expression of the reference gene, 18 S ribosomal RNA (rRNA). The conditions for qRT-PCR began with an initial activation step of heating at 95 °C for 5 min. This was followed by amplification phase comprising of 45 cycles of denaturation at 94 °C for 20 s, annealing at 55 °C for 20 s, and extension at 72 °C for 15 s^74^. Data acquisition was performed using CFX Maestro™ Software (Bio-Rad, USA). The relative expression levels of cyclin D1, c-Myc and MDR1 were quantified in relation to the expression of the reference gene 18 S rRNA within the same sample by calculating and normalizing the threshold cycles (Ct) values of target genes against those of 18 S rRNA using ΔΔ^Ct^ method.

**Table 1:** primer sequence of cyclin D1, c-Myc, MDR1, and 18 S rRNA for qRT-PCR:

Gene	Primer sequence
Cyclin D1	F: 5′-CTTACTTCAAGTGCGTGCAGAGG-3′ R: 5′-GCTTGTTCACCAGAAGCAGTTCC-3′
C-Myc	F: 5′-AGGAACTATGACCTCGACTACG-3′ R: 5′-AGTAGCTCGGTCATCATCTCCAG-3′
MDR1	F: 5′-CCCATCATTGCAATAGCAGG-3′ R: 5′-TGTTCAAACTTCTGCTCCTGA-3′
18 S rRNA	F: 5′-GTAACCCGTTGAACCCCATT-3′ R: 5′-CAAGCTTATGACCCGCACTT-3′

### Histopathological examination

Liver tissues were preserved in 10% formalin, then embedded in paraffin wax, and cut into 5 μm sections. For histopathological examination, the slides were stained using hematoxylin and eosin stain (H&E).

To ensure objectivity and minimize observer bias, the histopathological examination was conducted using a blinded assessment protocol^75^. All tissue slides were coded to conceal the identity of the experimental groups. The pathologist performed the microscopic examination and recorded the descriptive findings without prior knowledge of the specific treatment received by each animal.

### Statistical analysis

Data was fed into a computer and analyzed using IBM SPSS software version 20.0. **(**Armonk, NY: IBM Corp**)**. Quantitative data were summarized using mean and standard deviation (SD) for all studied parameters. The significance of the results was assessed at 0.05 level. For normally distributed quantitative variables, an F-test (ANOVA) was used to compare more than two groups, and a Post Hoc test (Tukey) was used for pairwise comparisons.

## Conclusion

Based on discussion above, our study demonstrates that ATX possesses promising therapeutic potential in rats with DEN/CCl_4_-induced HCC. These effects likely stem from a variety of interlinked mechanisms, such as enhancing GSH levels, mitigating oxidative stress. Crucially, we provide evidence that combining ATX with DOX exerts a synergistic antitumor effect, primarily driven by the upregulation of GSK3β and the subsequent suppression of the oncogenic Wnt/β-catenin pathway. A pivotal finding of this work is the ability of ATX to modulate MDR1 expression, effectively reversing acquired chemoresistance and sensitizing HCC cells to chemotherapy. Consequently, these findings establish ATX as a promising adjuvant therapy that not only potentiates the cytotoxic efficacy of DOX but also overcomes its limitations regarding drug resistance.

## Supplementary Information

Below is the link to the electronic supplementary material.

Supplementary Material 1

## Data Availability

All data supporting the findings of this study are included within the manuscript. No additional datasets have been deposited in a public repository.

## References

[1] Holah, N. S., El-Azab, D. S., Aiad, H. A. & Sweed, D. M. Hepatocellular carcinoma in Egypt: Epidemiological and histopathological properties. *Menoufia Med. J.* **28** (4), 718–724. 10.4103/1110-2098.167895 (2015).

[2] Rashed, W. M., Kandeil, M. A. M., Mahmoud, M. O. & Ezzat, S. Hepatocellular carcinoma (HCC) in Egypt: A comprehensive overview. *J. Egypt. Natl. Cancer Inst.* **32** (1), 1–11. 10.1186/s43046-020-0016-x (2020). 10.1186/s43046-020-0016-x PMC13325438 32372179

[3] Rivankar, S. An overview of doxorubicin formulations in cancer therapy. *J. Cancer Res. Ther.* **10** (4), 853–858. 10.4103/0973-1482.139267 (2014). 25579518 10.4103/0973-1482.139267

[4] Yang, F., Teves, S. S., Kemp, C. J. & Henikoff, S. Doxorubicin, DNA torsion, and chromatin dynamics. *Biochim. Biophys. Acta Rev. Cancer*. **1845** (1), 84–89. 10.1016/j.bbcan.2013.12.002 (2014). 10.1016/j.bbcan.2013.12.002 PMC3927826 24361676

[5] Abdel-Hamid, N. M., Abass, S. A., Mohamed, A. A. & Hamid, D. M. Herbal management of hepatocellular carcinoma through cutting the pathways of the common risk factors. *Biomed. Pharmacother*. **107**, 1246–1258. 10.1016/j.biopha.2018.08.104 (2018). 30257339 10.1016/j.biopha.2018.08.104 PMC7127621

[6] Sadek, K., Abouzed, T., Nasr, S., Shoukry, M. & Licochalcone, B. Ameliorates liver cancer via targeting of apoptotic genes, DNA repair systems, and cell cycle control. *Iran. J. Pharm. Res.* **19** (4), 372–386 (2020). 33841550 10.22037/ijpr.2020.1101292 PMC8019863

[7] Sadek, K., Beltagy, D., Saleh, E. & Abouelkhair, R. Camel milk and bee honey regulate pro-fibrotic cytokine gene transcripts in carbon tetrachloride-induced liver cirrhosis. *Can. J. Physiol. Pharmacol.* **94** (11), 1141–1150 (2016). 27455095 10.1139/cjpp-2015-0596

[8] Krutsenko, Y., Singhi, A. D. & Monga, S. P. β-Catenin activation in hepatocellular cancer: Implications in biology and therapy. *Cancers* **3** (8), 1830. 10.3390/cancers13081830 (2021). 10.3390/cancers13081830 PMC8069637 33921282

[9] He, S. & Tang, S. WNT β-catenin signaling in the development of liver cancers. *Biomed. Pharmacother*. **132**, 110851. 10.1016/j.biopha.2020.110851 (2020). 33080466 10.1016/j.biopha.2020.110851

[10] Suarez, I. et al. Wnt/β-catenin signaling pathway in hepatocellular carcinomas cases from Colombia. *Ann. Hepatol.* **14** (1), 64–74. 10.1016/S1665-2681(19)30802-6 (2015). 25536643

[11] Medhi, J., Kalita, M. C. & Astaxanthin An algae-based natural compound with a potential role in human health-promoting effect: An updated comprehensive review. *J. Appl. Biol. Biotechnol.* **9** (1), 114–123. 10.7324/JABB.2021.9115 (2021).

[12] Li, J., Guo, C. & Wu, J. Astaxanthin in liver health and disease: A potential therapeutic agent. *Drug Des. Dev. Ther.* **14**, 2275–2285. 10.2147/DDDT.S230749 (2020). 10.2147/DDDT.S230749 PMC7293384 32606597

[13] Ambati, R. R., Phang, S. M., Ravi, S., Aswathanarayana, R. G. & Astaxanthin Sources, extraction, stability, biological activities and its commercial applications - A review. *Mar. Drugs*. **12** (1), 128–152. 10.3390/md12010128 (2014). 24402174 10.3390/md12010128 PMC3917265

[14] Pereira, C. P. M., Souza, A. C. R., Vasconcelos, A. R. & Prado, P. S. Antioxidant and anti-inflammatory mechanisms of action of astaxanthin in cardiovascular diseases. *Int. J. Mol. Med.* **47** (1), 37–48. 10.3892/ijmm.2020.4783 (2021). 33155666 10.3892/ijmm.2020.4783 PMC7723678

[15] Zhang, L. & Wang, H. Multiple mechanisms of anti-cancer effects exerted by astaxanthin. *Mar. Drugs*. **13** (7), 4310–4330. 10.3390/md13074310 (2015). 26184238 10.3390/md13074310 PMC4515619

[16] Faraone, I. et al. Astaxanthin anticancer effects are mediated through multiple molecular mechanisms: A systematic review. *Pharmacol. Res.* **155**, 104689. 10.1016/j.phrs.2020.104689 (2020). 32057895 10.1016/j.phrs.2020.104689

[17] Li, J. et al. Astaxanthin inhibits proliferation and induces apoptosis of human hepatocellular carcinoma cells via inhibition of of Nf-Κb P65 and Wnt/Β-catenin in vitro. *Mar. Drugs*. **13** (10), 6064–6081. 10.3390/md13106064 (2015). 26404320 10.3390/md13106064 PMC4626679

[18] Jung, J. Human tumor xenograft models for preclinical assessment of anticancer drug development. *Toxicol. Res.* **30** (1), 1–5. 10.5487/TR.2014.30.1.001 (2014). 24795792 10.5487/TR.2014.30.1.001 PMC4007037

[19] Blumer, T. et al. Hepatocellular carcinoma xenografts established from needle biopsies preserve the characteristics of the originating tumors. *Hepatol. Commun.* **3** (7), 971–986. 10.1002/hep4.1365 (2019). 31334445 10.1002/hep4.1365 PMC6601318

[20] Uehara, T., Pogribny, I. P. & Rusyn, I. The DEN and CCl4-induced mouse model of fibrosis and inflammation‐associated hepatocellular carcinoma. *Curr. Protoc.*10.1002/cpz1.211 (2021). 10.1002/cpz1.211 PMC8744072 34370903

[21] Sengupta, D., Chowdhury, K. D., Sarkar, A., Paul, S. & Sadhukhan, G. C. Berberine and S allyl cysteine mediated amelioration of DEN + CCl4 induced hepatocarcinoma. *Biochim. Biophys. Acta Gen. Subj.* **1840** (1), 219–244. 10.1016/j.bbagen.2013.08.020 (2014). 10.1016/j.bbagen.2013.08.020 23999088

[22] Sayed, E. L., Morsy, H. E., Abo, L. E., Emara, T. M. & Galhom, R. A. Effect of carbon tetrachloride (CCL4) on liver in adult albino rats: Histological study. *Egypt. J. Hosp. Med.* **76** (6), 4254–4261. 10.21608/ejhm.2019.43804 (2019).

[23] Bakiri, L. & Wagner, E. F. Mouse models for liver cancer. *Mol. Oncol.* **7** (2), 206–223. 10.1016/j.molonc.2013.01.005 (2013). 23428636 10.1016/j.molonc.2013.01.005 PMC5528415

[24] Santos, N. P., Colaço, A. A. & Oliveira, P. A. Animal models as a tool in hepatocellular carcinoma research: A Review. *Tumor Biol.* **39** (3), 1010428317695923. 10.1177/1010428317695923 (2017). 10.1177/1010428317695923 28347231

[25] Ohno, T. et al. Preventive effects of astaxanthin on diethylnitrosamine-induced liver tumorigenesis in C57/BL/KsJ-db/db obese mice. *Hepatol. Res.* **46** (3), E201–E209. 10.1111/hepr.12550 (2016). 26147624 10.1111/hepr.12550

[26] Alansari, W. S. & Eskandrani, A. A. The anticarcinogenic effect of the apple polyphenol phloretin in an experimental rat model of hepatocellular carcinoma. *Arab. J. Sci. Eng.* **45** (6), 4589–4597. 10.1007/s13369-020-04478-7 (2020).

[27] Zeweil, M. M. et al. Sidr honey abrogates the oxidative stress and downregulates the hyaluronic acid concentration and gene expression of TGF- β 1 and COL1a1 in rat model of thioacetamide-induced hepatic fibrosis. *Anim. Sci. J.* **91**, e13434 (2020). 32696560 10.1111/asj.13434

[28] Metibemu, D. S. et al. Carotenoid isolates of Spondias mombin demonstrate anticancer effects in DMBA-induced breast cancer in Wistar rats through X-linked inhibitor of apoptosis protein (XIAP) antagonism and anti-inflammation. *J. Food Biochem.* **44** (12), e13523. 10.1111/jfbc.13523 (2020). 33084091 10.1111/jfbc.13523

[29] Turkez, H., Geyikoglu, F. & Yousef, M. I. Beneficial effect of astaxanthin on 2,3,7,8-tetrachlorodibenzo-p-dioxin-induced liver injury in rats. *Toxicol. Ind. Health*. **29** (7), 591–599. 10.1177/0748233711434959 (2013). 22312033 10.1177/0748233711434959

[30] Jasim, S. M. & Jwad, S. M. Astaxanthin extract potentiates the functional performance of hepatic and renal tissues and can prevent or alleviate hepatotoxicity and nephrotoxicity induced by acetaminophen. *Ann. Rom Soc. Cell. Biol.* **25** (3), 560–568 (2021).

[31] Lebda, M. A., Sadek, K. M., Abouzed, T. K., Tohamy, H. G. & El-Sayed, Y. S. Melatonin mitigates thioacetamide-induced hepatic fi brosis via antioxidant activity and modulation of proin fl ammatory cytokines and fi brogenic genes. *Life Sci.* **192**, 136–143 (2018). 29180002 10.1016/j.lfs.2017.11.036

[32] Xue, Y. et al. Downregulation of Frizzled-7 induces the apoptosis of hepatocellular carcinoma cells through inhibition of NF-κB. *Oncol. Lett.* **15** (5), 7693–7701. 10.3892/ol.2018.8292 (2018). 29731900 10.3892/ol.2018.8292 PMC5920807

[33] Deldar Abad Paskeh, M., Mirzaei, S., Ashrafizadeh, M., Zarrabi, A. & Sethi, G. Wnt / β -Catenin signaling as a driver of hepatocellular carcinoma progression: An emphasis on molecular pathways. *J. Hepatocell Carcinoma*. **8**, 1415–1444. 10.2147/JHC.S336858 (2021). 34858888 10.2147/JHC.S336858 PMC8630469

[34] Copat, C. et al. Astaxanthin in cancer therapy and prevention. *Biomed. Rep.* **22** (4), 66. 10.3892/br.2025.1944 (2025). 40017498 10.3892/br.2025.1944 PMC11865706

[35] Tung, E. K. K., Wong, B. Y. C., Yau, T. O. & Ng, I. O. L. Upregulation of the Wnt co-receptor LRP6 promotes hepatocarcinogenesis and enhances cell invasion. *PLoS One*. **7** (5), 1–10. 10.1371/journal.pone.0036565 (2012). 10.1371/journal.pone.0036565 PMC3343020 22570728

[36] Preziosi, M., Poddar, M., Singh, S. & Monga, S. P. Hepatocyte wnts are dispensable during diethylnitrosamine and carbon tetrachloride-induced injury and hepatocellular cancer. *Gene Expr*. **18** (3), 209–219. 10.3727/105221618X15205148413587 (2018). 29519268 10.3727/105221618X15205148413587 PMC6190118

[37] Mccubrey, J. A. et al. GSK-3 as potential target for therapeutic intervention in cancer. *Oncotarget* **5** (10), 2881–2911. 10.18632/oncotarget.2037 (2014). 24931005 10.18632/oncotarget.2037 PMC4102778

[38] Ban, K. C., Singh, H., Krishnan, R. & Seow, H. F. GSK-3β phosphorylation and alteration of β-catenin in 8390hepatocellular carcinoma. *Cancer Lett.* **199** (2), 201–208. 10.1016/S0304-3835(03)00421-X (2003). 12969793 10.1016/s0304-3835(03)00421-x

[39] Kowshik, J. et al. Astaxanthin inhibits hallmarks of cancer by targeting the PI3K/NF-κΒ/STAT3 signalling axis in oral squamous cell carcinoma models. *IUBMB Life*. **71** (10), 1595–1610. 10.1002/iub.2104 (2019). 31251469 10.1002/iub.2104

[40] Saleh, D. O., Mahmoud, S. S., Hassan, A. & Sanad, E. F. Doxorubicin-induced hepatic toxicity in rats: Mechanistic protective role of Omega-3 fatty acids through Nrf2/HO-1 activation and PI3K/Akt/GSK-3β axis modulation. *Saudi J. Biol. Sci.* **29** (7), 103308. 10.1016/j.sjbs.2022.103308 (2022). 35677895 10.1016/j.sjbs.2022.103308 PMC9167977

[41] MacDonald, B. T., Tamai, K. & He, X. Wnt/β-catenin signaling: Components, mechanisms, and diseases. *Dev. Cell.* **17** (1), 9–26. 10.1016/j.devcel.2009.06.016 (2009). 19619488 10.1016/j.devcel.2009.06.016 PMC2861485

[42] Zarneshan, S. N., Fakhri, S., Farzaei, M. H., Khan, H. & Saso, L. Astaxanthin targets PI3K/Akt signaling pathway toward potential therapeutic applications. *Food Chem. Toxicol.* **145**, 111714. 10.1016/j.fct.2020.111714 (2020). 32871194 10.1016/j.fct.2020.111714

[43] Musgrove, E. A., Caldon, C. E., Barraclough, J., Stone, A. & Sutherland, R. L. Cyclin D as a therapeutic target in cancer. *Nat. Rev. Cancer*. **11** (8), 558–572. 10.1038/nrc3090 (2011). 21734724 10.1038/nrc3090

[44] Zhang, Y. et al. Chemotherapeutic drugs induce oxidative stress associated with DNA repair and metabolism modulation. *Life Sci.* **289**, 120242. 10.1016/j.lfs.2021.120242 (2022). 34922939 10.1016/j.lfs.2021.120242

[45] Deguchi, T. & Pitot, H. C. Expression of c-myc in altered hepatic foci induced in rats by various single doses of diethylnitrosamine and promotion by 0.05% phenobarbital. *Mol. Carcinog.* **14** (3), 152–159. 10.1002/mc.2940140304 (1995). 7576107 10.1002/mc.2940140304

[46] He, T. C. et al. Identification of c-MYC as a target of the APC pathway. *Science* **281** (5382), 1509–1512. 10.1126/science.281.5382.1509 (1998). 9727977 10.1126/science.281.5382.1509

[47] Akita, H. et al. MYC activates stem-like cell potential in hepatocarcinoma by a p53-dependent mechanism. *Cancer Res.* **74** (20), 5903–5913. 10.1158/0008-5472.CAN-14-0527 (2014). 25189530 10.1158/0008-5472.CAN-14-0527 PMC4199878

[48] Kuo, M. T. Redox regulation of multidrug resistance in cancer chemotherapy: Molecular mechanisms and therapeutic opportunities. *Antioxid. Redox Signal.* **11** (1), 99–133. 10.1089/ars.2008.2095 (2009). 18699730 10.1089/ars.2008.2095 PMC2577715

[49] Kaluç, N. & Thomas, P. B. Astaxanthin provides cytoprotection in response to oxidative stress in an autophagy-dependent manner. *J. Clin. Pract. Res.* **47** (4), 407–414. 10.14744/cpr.2025.27738 (2025). 41257064 10.14744/cpr.2025.27738 PMC12478580

[50] Housman, G. et al. Drug resistance in cancer: An overview. *Cancers* **6** (3), 1769–1792. 10.3390/cancers6031769 (2014). 25198391 10.3390/cancers6031769 PMC4190567

[51] Gottesman, M. M., Fojo, T. & Bates, S. E. Multidrug resistance in cancer: Role of ATP-dependent transporters. *Nat. Rev. Cancer*. **2** (1), 48–58. 10.1038/nrc706 (2002). 11902585 10.1038/nrc706

[52] Ma, H. et al. Astaxanthin from: Haematococcus pluvialis ameliorates the chemotherapeutic drug (doxorubicin) induced liver injury through the Keap1/Nrf2/HO-1 pathway in mice. *Food Funct.* **11** (5), 4659–4671. 10.1039/C9FO02429H (2020). 32405635 10.1039/c9fo02429h

[53] Islam, M. A. et al. Astaxanthin Ameliorates Hepatic Damage and Oxidative Stress in Carbon Tetrachloride–administered Rats. *Pharmacogn Res.*10.4103/pr.pr_26_17 (2017). 10.4103/pr.pr_26_17 PMC5757332 29333048

[54] Shibata, A., Kiba, Y., Akati, N., Fukuzawa, K. & Terada, H. Molecular characteristics of astaxanthin and β-carotene in the phospholipid monolayer and their distributions in the phospholipid bilayer. *Chem. Phys. Lipids*. **113** (1–2), 11–22. 10.1016/S0009-3084(01)00136-0 (2001). 11687223 10.1016/s0009-3084(01)00136-0

[55] Wei, Y. et al. Remarkable protective effects of Nrf2-mediated antioxidant enzymes and tissue specificity in different skeletal muscles of daurian ground squirrels over the torpor-arousal cycle. *Front. Physiol.* **10**, 1449. 10.3389/fphys.2019.01449 (2019). 31824343 10.3389/fphys.2019.01449 PMC6883408

[56] Bingül, I. et al. Blueberry treatment attenuated cirrhotic and preneoplastic lesions and oxidative stress in the liver of diethylnitrosamine-treated rats. *Int. J. Immunopathol. Pharmacol.* **29** (3), 426–437. 10.1177/0394632015621319 (2016). 26684621 10.1177/0394632015621319 PMC5806765

[57] Atwa, G., Omran, G., Elbaky, A. A. & Okda, T. The antitumour effect of galangin and luteolin with doxorubicin on chemically induced hepatocellular carcinoma in rats. *Contemp. Oncol. /Współczesna Onkol*. **25** (3), 174–184. 10.5114/wo.2021.110048 (2021). 10.5114/wo.2021.110048 PMC8547180 34729037

[58] Lee, K. T. et al. Glutathione status in the blood and tissues of patients with virus-originated hepatocellular carcinoma. *Clin. Biochem.* **40** (15), 1157–1162. 10.1016/j.clinbiochem.2007.06.012 (2007). 17706189 10.1016/j.clinbiochem.2007.06.012

[59] Tsai, S. M. et al. Evaluation of redox statuses in patients with hepatitis B virus-associated hepatocellular carcinoma. *Ann. Clin. Biochem.* **46** (5), 394–400. 10.1258/acb.2009.009029 (2009). 19641006 10.1258/acb.2009.009029

[60] Mossenta, M., Busato, D., Dal Bo, M. & Toffoli, G. Glucose metabolism and oxidative stress in hepatocellular carcinoma: Role and possible implications in novel therapeutic strategies. *Cancers* **12** (6), 1668. 10.3390/cancers12061668 (2020). 32585931 10.3390/cancers12061668 PMC7352479

[61] Lin, C. C. & Yin, M. C. B vitamins deficiency and decreased anti-oxidative state in patients with liver cancer. *Eur. J. Nutr.* **46** (5), 293–299. 10.1007/s00394-007-0665-8 (2007). 17571208 10.1007/s00394-007-0665-8

[62] Yahya, R. S., Ghanem, O. H., Foyouh, A. A., Atwa, M. A. & Enany, S. A. Role of interleukin-8 and oxidative stress in patients with hepatocellular carcinoma. *Clin. Lab.* **59** (9–10), 969–976. 10.7754/clin.lab.2012.120712 (2013). 24273918 10.7754/clin.lab.2012.120712

[63] Funato, Y., Michiue, T., Asashima, M. & Miki, H. The thioredoxin-related redox-regulating protein nucleoredoxin inhibits Wnt – β -catenin signalling through Dishevelled. *Nat. Cell. Biol.* **8** (5), 501–508. 10.1038/ncb1405 (2006). 16604061 10.1038/ncb1405

[64] Workman, P. et al. Guidelines for the welfare and use of animals in cancer research. *Br. J. Cancer*. **102** (11), 1555–1577. 10.1038/sj.bjc.6605642 (2010). 20502460 10.1038/sj.bjc.6605642 PMC2883160

[65] Percie du Sert, N. et al. The ARRIVE guidelines 2.0: Updated guidelines for reporting animal research. *JCBFM* **40** (9), 1769–1777. 10.1177/0271678X20943823 (2020). 10.1177/0271678X20943823 PMC7430098 32663096

[66] Motawi, T. K., El-Boghdady, N. A., El-Sayed, A. M. & Helmy, H. S. Comparative study of the effects of PEGylated interferon-α2a versus 5-fluorouracil on cancer stem cells in a rat model of hepatocellular carcinoma. *Tumor Biol.* **37** (2), 1617–1625. 10.1007/s13277-015-3920-2 (2016). 10.1007/s13277-015-3920-2 26304505

[67] Golla, K., Cherukuvada, B., Ahmed, F., Kondapi, A. K. & Efficacy Safety and Anticancer Activity of Protein Nanoparticle-Based Delivery of Doxorubicin through Intravenous Administration in Rats. *PloS One*. 10.1371/journal.pone.0051960 (2012). e51960. 10.1371/journal.pone.0051960 PMC3528733 23284832

[68] Tripathi, D. N. & Jena, G. B. Astaxanthin intervention ameliorates cyclophosphamide-induced oxidativestress, DNA damage and early hepatocarcinogenesis in rat: Role of Nrf2, p53, p38 and phase-II enzymes. *Mutat. Res. Genet. Toxicol. Environ. Mutagen.* **696** (1), 69–80. 10.1016/j.mrgentox.2009.12.014 (2010). 10.1016/j.mrgentox.2009.12.014 20038455

[69] El-Naggar, N. E. A. et al. Artificial neural network approach for prediction of AuNPs biosynthesis by Streptomyces flavolimosus, characterization, antitumor potency in-vitro and in-vivo against Ehrlich ascites carcinoma. *Sci. Rep.* **13** (1), 12686. 10.1038/s41598-023-39177-4 (2023). 37542154 10.1038/s41598-023-39177-4 PMC10403537

[70] Griffith, O. W. Determination of glutathione and glutathione disulfide using glutathione reductase and 2-vinylpyridine. *Anal. Biochem.* **106** (1), 207–212. 10.1016/0003-2697(80)90139-6 (1980). 7416462 10.1016/0003-2697(80)90139-6

[71] Draper, H. H. & Hadley, M. Malondialdehyde determination as index of lipid Peroxidation. *Methods Enzymol.* **186**, 421–431. 10.1016/0076-6879(90)86135-I (1990). 2233309 10.1016/0076-6879(90)86135-i

[72] Lowery, O. H., Rosebrough, N. J., Farr, A. L. & Randall, R. J. Protein measurement with the Folin phenol reagent. *J. Biol. Chem.* **193** (1), 265–275. 10.1016/S0021-9258(19)52451-6 (1951). 14907713

[73] Harlow, E. & Lane, D. *Using antibodies: A laboratory manual* (CSHL, 1999).

[74] Gowayed, M., Mahmoud, S., El–Sayed, Y., Abu Samra, N. & Kamel, M. Enhanced mitochondrial biogenesis is associated with the ameliorative action of creatine supplementation in rat soleus and cardiac muscles. *Exp. Ther. Med.* **19** (1), 384–392. 10.3892/etm.2019.8173 (2020). 31853315 10.3892/etm.2019.8173 PMC6909667

[75] Gibson-corley, K. N., Olivier, A. K. & Meyerholz, D. K. Principles for valid histopathologic scoring in research. *Vet. Pathol.* **50** (6), 1007–1015. 10.1177/0300985813485099 (2013). 23558974 10.1177/0300985813485099 PMC3795863

