# Supplementary material for: Astaxanthin suppresses hepatocellular carcinoma via targeting Wnt/Β-catenin pathway: Experimental study on chemically induced HCC in rats
Source: Sci Rep. 2026 Apr 21;16:12928. doi: 10.1038/s41598-026-45680-1 (PMC13096522; doi:10.1038/s41598-026-45680-1)
Supplement: Supplementary file 1 — Supplementary Material 1 [file 41598_2026_45680_MOESM1_ESM.docx]

**Supplementary Information for *“*Astaxanthin suppresses hepatocellular carcinoma via targeting Wnt/Β-catenin pathway: Experimental study on chemically induced HCC in rats*”***

Mona A. Kortam ^a^, Marwa Sh. Ismail ^a,b*^, Maher A. Kamel ^c^, Noha A. El-Boghdady ^a^

^a Department of Biochemistry, Faculty of Pharmacy, Cairo University, 23 Kasr El-Aini St., Cairo 11562, Egypt.^

^b Department of Pharmacology and Therapeutics, Faculty of Pharmacy, Pharos University in Alexandria (PUA), Canal El Mahmoudia Street, beside the Green Plaza Complex, Alexandria 21648, Egypt.^

^c Department of Biochemistry, Medical Research Institute, Alexandria University, 165 Al-Horreya Avenue, Hadara, Alexandria^ ^21561, Egypt.^


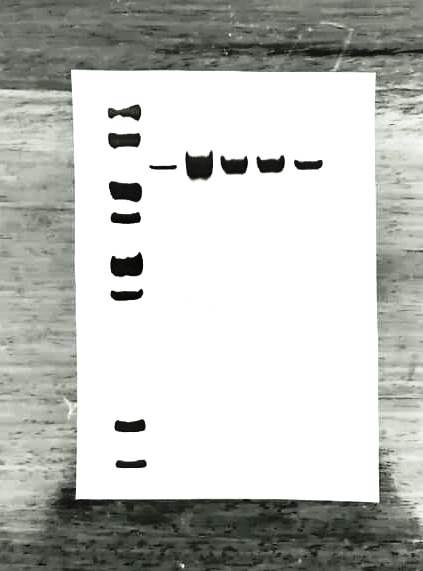


22

28

50

38

113

78

62

154

**ATX+DOX**

**ATX**

**Control**

**HCC**

**DOX**

**Supplementary Figure S1** Full-length gel FZD-7 western blot image used for quantification in Fig 2c, showing protein expression in liver tissues from the control, HCC, ATX-treated, DOX-treated, and ATX+DOX-treated groups. β-actin used as the loading control. All lanes were run on the same gel and detected in a single exposure.


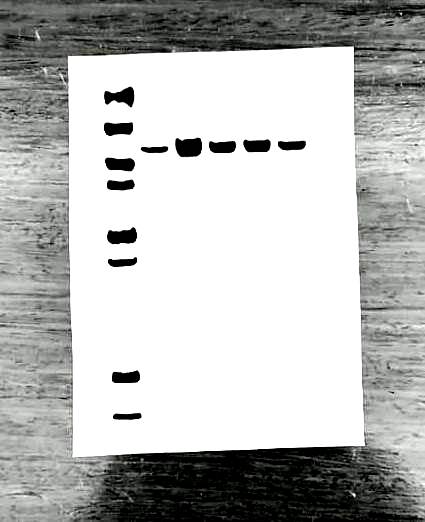


22

28

38

50

78

62

154

113

**HCC**

**ATX**

**DOX**

**ATX+DOX**

**Control**

**Supplementary Figure S2** Full-length gel FZD-7 western blot image used for quantification in Fig 2c, showing protein expression in liver tissues from the control, HCC, ATX-treated, DOX-treated, and ATX+DOX-treated groups. β-actin used as the loading control. All lanes were run on the same gel and detected in a single exposure.


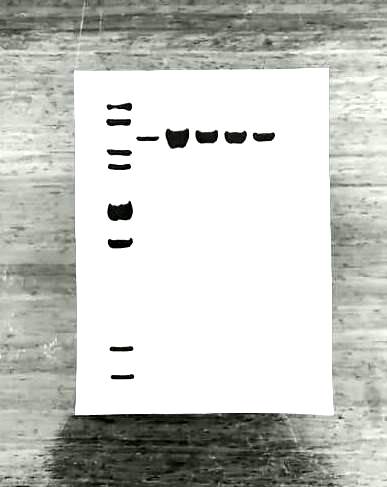


**ATX+DOX**

**DOX**

154

113

62

78

50

38

22

28

**ATX**

**HCC**

**Control**

**Supplementary Figure S3** Full-length gel FZD-7 western blot image used for quantification in Fig 2c, showing protein expression in liver tissues from the control, HCC, ATX-treated, DOX-treated, and ATX+DOX-treated groups. β-actin used as the loading control. All lanes were run on the same gel and detected in a single exposure.


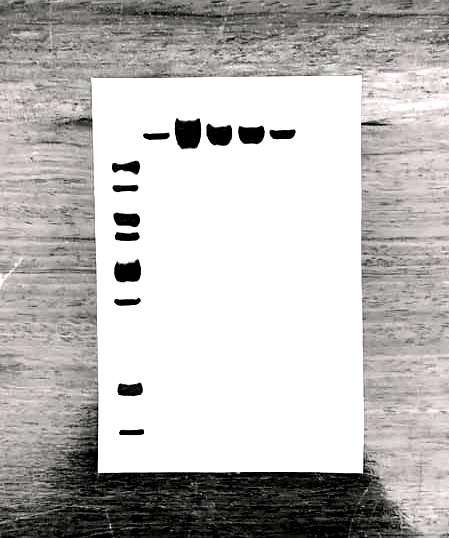


38

50

78

62

113

154

22

28

50

**DOX**

**Control**

**HCC**

**ATX+DOX**

**ATX**

**Supplementary Figure S4** Full-length gel LRP5/6 western blot image used for quantification in Fig 2d, showing protein expression in liver tissues from the control, HCC, ATX-treated, DOX-treated, and ATX+DOX-treated groups. β-actin used as the loading control. All lanes were run on the same gel and detected in a single exposure.


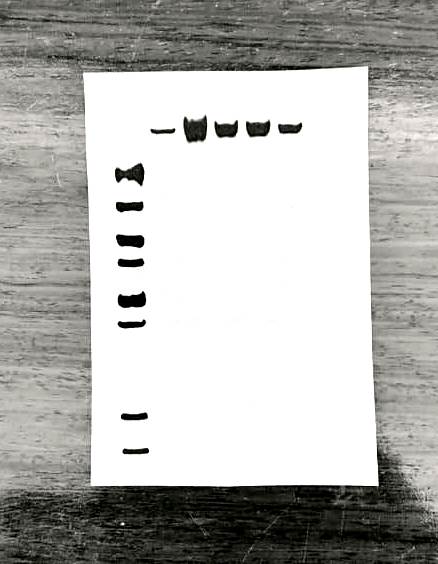


28

22

38

50

62

78

113

154

154

78

113

78

**ATX+DOX**

**DOX**

**ATX**

**HCC**

**Control**

**Supplementary Figure S5** Full-length gel LRP5/6 western blot image used for quantification in Fig 2d, showing protein expression in liver tissues from the control, HCC, ATX-treated, DOX-treated, and ATX+DOX-treated groups. β-actin used as the loading control. All lanes were run on the same gel and detected in a single exposure.


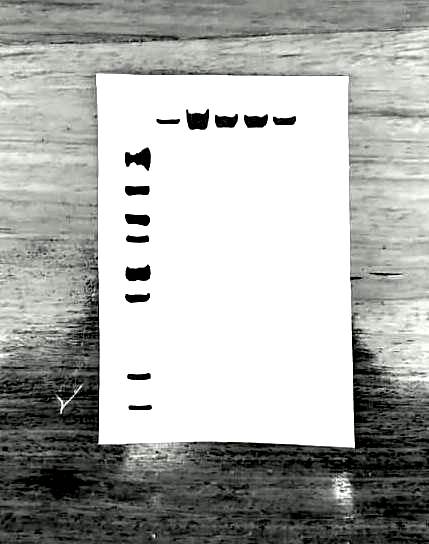


154

22

28

78

62

50

38

113

**Control**

**DOX**

**ATX+DOX**

**ATX**

**HCC**

**Supplementary Figure S6** Full-length gel LRP5/6 western blot image used for quantification in Fig 2d, showing protein expression in liver tissues from the control, HCC, ATX-treated, DOX-treated, and ATX+DOX-treated groups. β-actin used as the loading control. All lanes were run on the same gel and detected in a single exposure.

**
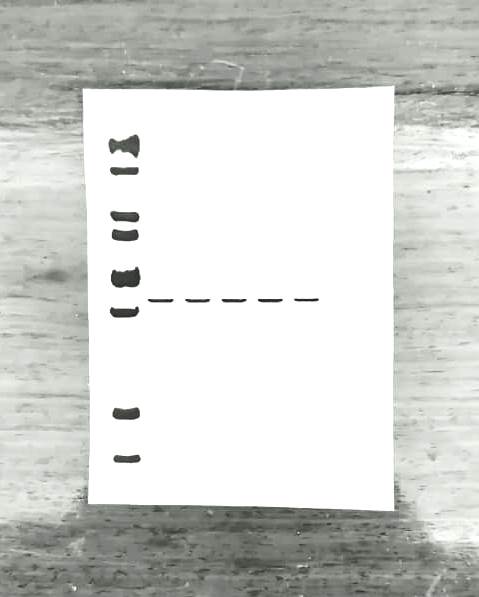
**

**HCC**

22

28

38

50

62

78

154

113

**DOX**

**ATX+DOX**

**ATX**

**Control**

**Supplementary Figure S7** Full-length gel β-actin western blot image used for quantification in Figs 2c and 2d, showing protein expression in liver tissues from the control, HCC, ATX-treated, DOX-treated, and ATX+DOX-treated groups. All lanes were run on the same gel and detected in a single exposure.


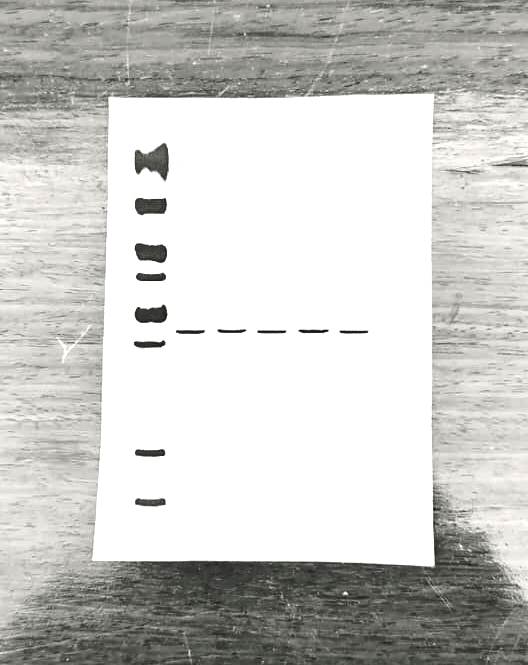


154

113

78

62

50

38

28

22

**DOX**

**Control**

**ATX**

**ATX+DOX**

**HCC**

**Supplementary Figure S8** Full-length gel β-actin western blot image used for quantification in Figs 2c and 2d, showing protein expression in liver tissues from the control, HCC, ATX-treated, DOX-treated, and ATX+DOX-treated groups. All lanes were run on the same gel and detected in a single exposure.


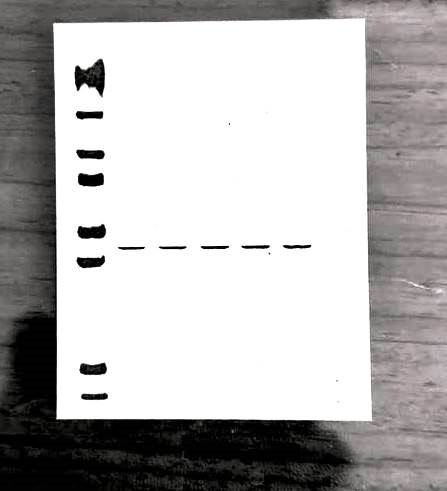


113

154

78

62

50

38

28

22

113

**DOX**

**ATX+DOX**

**HCC**

**ATX**

**Control**

**Supplementary Figure S9** Full-length gel β-actin western blot image used for quantification in Figs 2c and 2d, showing protein expression in liver tissues from the control, HCC, ATX-treated, DOX-treated, and ATX+DOX-treated groups. All lanes were run on the same gel and detected in a single exposure.
